# Estimating severity of influenza epidemics from severe acute respiratory infections (SARI) in intensive care units

**DOI:** 10.1186/s13054-018-2274-8

**Published:** 2018-12-19

**Authors:** Liselotte van Asten, Angie Luna Pinzon, Dylan W. de Lange, Evert de Jonge, Frederika Dijkstra, Sierk Marbus, Gé A. Donker, Wim van der Hoek, Nicolette F. de Keizer

**Affiliations:** 1 Centre for Infectious Disease Control Netherlands, National Institute for Public Health and the Environment (RIVM), Bilthoven, The Netherlands; 2 National Intensive Care Evaluation, Amsterdam, the Netherlands; 3 Department of Intensive Care Medicine, University Medical Center, Utrecht University, Utrecht, Netherlands; 4 Department of Intensive Care, Leiden University Medical Center, Leiden, the Netherlands; 5 Nivel Primary Care Database – Sentinel Practices, Utrecht, the Netherlands; 6 Department of Medical Informatics, Amsterdam UMC, Location AMC, Amsterdam Public Health Research Institute, Amsterdam, The Netherlands

**Keywords:** Severe acute respiratory infections, SARI, Intensive care, Influenza, Pneumonia, Severity, Burden

## Abstract

**Background:**

While influenza-like-illness (ILI) surveillance is well-organized at primary care level in Europe, few data are available on more severe cases. With retrospective data from intensive care units (ICU) we aim to fill this current knowledge gap. Using multiple parameters proposed by the World Health Organization we estimate the burden of severe acute respiratory infections (SARI) in the ICU and how this varies between influenza epidemics.

**Methods:**

We analyzed weekly ICU admissions in the Netherlands (2007–2016) from the National Intensive Care Evaluation (NICE) quality registry (100% coverage of adult ICUs in 2016; population size 14 million) to calculate SARI incidence, SARI peak levels, ICU SARI mortality, SARI mean Acute Physiology and Chronic Health Evaluation (APACHE) IV score, and the ICU SARI/ILI ratio. These parameters were calculated both yearly and per separate influenza epidemic (defined epidemic weeks). A SARI syndrome was defined as admission diagnosis being any of six pneumonia or pulmonary sepsis codes in the APACHE IV prognostic model. Influenza epidemic periods were retrieved from primary care sentinel influenza surveillance data.

**Results:**

Annually, an average of 13% of medical admissions to adult ICUs were for a SARI but varied widely between weeks (minimum 5% to maximum 25% per week). Admissions for bacterial pneumonia (59%) and pulmonary sepsis (25%) contributed most to ICU SARI. Between the eight different influenza epidemics under study, the value of each of the severity parameters varied. Per parameter the minimum and maximum of those eight values were as follows: ICU SARI incidence 558–2400 cumulated admissions nationwide, rate 0.40–1.71/10,000 inhabitants; average APACHE score 71–78; ICU SARI mortality 13–20%; ICU SARI/ILI ratio 8–17 cases per 1000 expected medically attended ILI in primary care); peak-incidence 101–188 ICU SARI admissions in highest-incidence week, rate 0.07–0.13/10,000 population).

**Conclusions:**

In the ICU there is great variation between the yearly influenza epidemic periods in terms of different influenza severity parameters. The parameters also complement each other by reflecting different aspects of severity. Prospective syndromic ICU SARI surveillance, as proposed by the World Health Organization, thereby would provide insight into the severity of ongoing influenza epidemics, which differ from season to season.

**Electronic supplementary material:**

The online version of this article (10.1186/s13054-018-2274-8) contains supplementary material, which is available to authorized users.

## Introduction

Though advocated by the World Health Organization (WHO) and the European Centre for Disease Prevention and Control (ECDC), surveillance of severe acute respiratory infections (SARI) that require hospital admission is implemented in only few Western European countries [1–4]. In contrast, surveillance of acute respiratory infections (ARI) or influenza-like illness (ILI) in primary care is well-established as part of the European Influenza Surveillance Network (EISN), coordinated by the ECDC [5, 6]. Such primary care surveillance covers the community dwelling population and is thus focused on patients with milder illness. The number of patients developing serious complications and who require hospitalization is not available through this system and therefore, our understanding of the burden of respiratory infections is incomplete. The WHO recommends development and application of additional SARI measures that can be used to assess the severity of every influenza epidemic, whether seasonal or pandemic, and creating a historical SARI baseline [7]. The proposed measures include: SARI incidence, SARI peak levels, SARI mortality, and the SARI/ILI ratio. A SARI is a so-called syndrome group, i.e. based on rapidly available (initial) diagnoses that can be monitored as an indicator of infectious disease trends, outbreaks, and burden [8].

The most important complication of influenza virus infection is pneumonia (primary viral or secondary bacterial pneumonia) [9]. While costs can be high, the precise burden remains a blind spot [10–15]. Such burden information is crucial for prevention and response considering that vaccination, the main control measure against influenza infection, is aimed at preventing complications. A severe influenza season may also lead to hospital capacity problems, especially in ICUs. In this study we analyze comprehensive retrospective ICU data to fill the current knowledge gap.

## Methods

We combined two databases (of ICU and ILI data) for the 2007–2016 time period.

### Intensive care data

We used data from the National Intensive Care Evaluation (NICE) registry, which has registered adult ICU admissions for quality monitoring purposes [16, 17] since 1996. The coverage has increased over time reaching 90% coverage in 2012 and nearly 100% in 2016. Since 2007, all ICUs participating in the NICE registry have adopted the Acute Physiology and Chronic Health Evaluation IV (APACHE IV) model, which has more detailed diagnostic categories than the previous APACHE II model [18, 19].

We used weekly data on number of participating ICUs, number of medical ICU admissions (i.e. excluding admissions for surgery or for trauma), number of ICU SARI admissions (total and per APACHE IV diagnosis code), mean score from the APACHE IV model, in-ICU mortality, and age group. APACHE scores are commonly used in ICUs to quantify the severity of the illness of the patients (the higher the score the more severely ill), and to adjust for severity of illness when comparing outcome (mortality, length of stay) of different populations of patients [19].

We defined a syndrome group for ICU SARI admissions by including any respiratory diagnosis that could potentially have been of an infectious origin, i.e. caused by a respiratory pathogen such as influenza. A patient was considered an ICU SARI admission when all three of the following criteria were met: (1) the patient was admitted to the hospital less than 2 days before ICU admission (to differentiate between community acquired versus nosocomial infection), (2) the ICU admission was not a readmission to the ICU within the hospitalized period, and (3) the APACHE IV reason for admission included any of the six respiratory codes for pulmonary sepsis or pneumonia (Table 2). Patients can have up to two APACHE IV codes registered upon admission. The NICE registry does not contain results of microbiological laboratory results.

### Influenza-like-illness data

Influenza surveillance in the Netherlands is based on sentinel general practitioners (GPs) participating in the Nivel Primary Care database, actively reporting the weekly number of patients consulting them for ILI [20]. ILI is defined as (1) sudden onset of symptoms, (2) fever, and (3) at least one of the following symptoms: cough, rhinorrhoea, sore throat, frontal headache, retrosternal pain, or myalgia [21]. GPs also take swabs from a random subset of patients with ILI for virological examination to determine whether influenza virus is circulating in the general population [20]. Epidemic weeks were those weeks in which the overall ILI incidence exceeded the threshold of 5.1/10,000 persons for a minimum of 2 consecutive weeks, which is based on the moving epidemic method used by countries participating in EISN [22] (the prospective data having small differences with fully completed retrospective datasets [23, 24]). During the nine seasons in the study period there were eight influenza epidemics of varying duration (based on all age groups combined). While influenza was circulating and ILI incidence crossed the threshold twice but not in consecutive weeks, retrospectively there was no influenza epidemic in 2011/2012. We further selected ILI in patients age 15 years and older, as only adult ICU admissions were available (15 years being the child/adult cutoff available in the ILI registry). We used season-years (which we defined as running from week 27 to week 26 in the next year) instead of calendar years, as respiratory pathogens circulate mostly in winter.

### Population size data

Data on the Dutch population size by age (1 January in each year) were extracted from the website of Statistics Netherlands (www.cbs.nl).

### Analyses

We described incidence of ICU SARI admissions during complete season-years and we described ICU SARI characteristics during defined influenza epidemics: incidence (cumulated over the epidemic weeks), rate (per 10,000 inhabitants), the contribution of the separate diagnoses to the syndrome, mean APACHE IV score, in-ICU SARI mortality, ICU SARI peak incidence (i.e. the highest registered weekly SARI incidence during an influenza epidemic), and the SARI/ILI ratio (the number of SARI per ILI case). All of these variables except for the APACHE score are proposed by the WHO. Characteristics were also stratified by age groups (15–44, 45–59, 60–64, and 65+ years). As the coverage increased over time, for comparison, we standardized the SARI incidences to the total number of medical ICU admissions in 2015/2016, when the NICE registry had near-complete national coverage. This was done by multiplying ICU SARI cases by the factor required to adjust the total number of medical admissions in each respective year to the total number of medical admissions observed in 2015/2016. The incidence of ILI was standardized to the size of the covered sentinel population of 2015/2016 (yearly coverage approximates 0.7% of the Dutch population, fluctuating slightly from year to year [20]). Due to the near-total coverage of the adult ICUs in 2015/2016 we used the standardized incidence and Dutch population-size data to calculate ICU SARI rates in the Dutch population. We estimated the ILI rates in the total adult Dutch population by extrapolating the coverage (~ 0.7%) to the total Dutch population size (yearly). Time series were depicted graphically and we calculated coefficients (Pearson chi-squared or Spearman’s rank where applicable) for correlation between time series.

## Results

We first present overall yearly characteristics of SARI patients (irrespective of influenza epidemic weeks); second, we provide SARI characteristics specifically per influenza epidemic.

### Overall ICU SARI characteristics irrespective of influenza epidemic periods

#### Total ICU SARI admissions (irrespective of influenza epidemics)

From January 2007 to December 2016 there were 588,571 registered ICU admissions, of which 257,589 (43.8%) had a medical reason for admission, of which 33,007 (13%) were SARI admissions in a total population size of 14 million adults in the Netherlands.

#### Year-to-year and week-to-week ICU SARI admissions (irrespective of influenza epidemics)

**Fig. 1:**
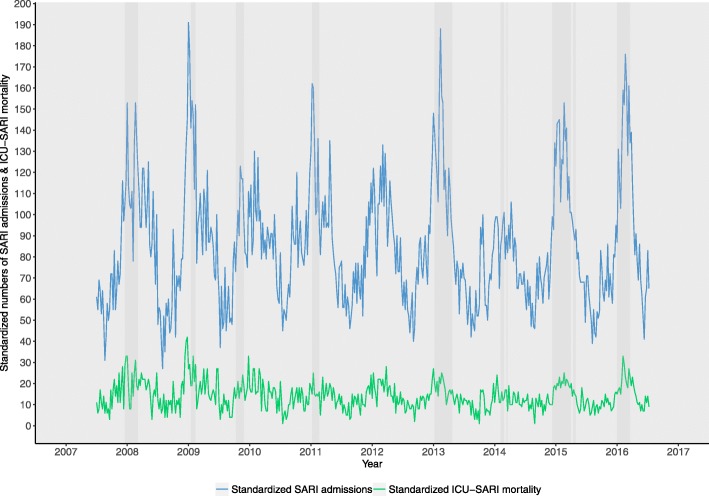
Standardized number of severe acute respiratory infections (SARI) admissions and standardized in-ICU SARI mortality per year (all ages combined) (grey shading: influenza epidemic weeks)

The overall proportion of ICU SARI admissions were relatively stable from one season-year to the next (11–14% of all ICU medical admissions were a SARI, Table 1), This proportion varied more from one week to the next (min 5% to max 25%) (standardized numbers are shown in Fig. 1, raw numbers in Additional file 1). The number of medical ICU admissions was higher when the number of ICU SARI admissions was higher (Spearman rank *R*^2^ 0.79, *p* < 0.001). The yearly rate of ICU SARI ranged from 2.74 to 3.35 per 10,000 inhabitants between the season-year (Table 1). The incidence was highest in the oldest age group (65+ years) and the proportion of ICU SARI admissions increased with increasing age (from an average 7% in 15–44-year-olds to 15% in the 65+ group). However, weekly, the highest proportion was in the 60–64 years age group reaching 36% in season 2015/2016.

**Table 1:** Number of ICUs in the NICE registration and medical and SARI admissions per season (2007–2016)

Season	Mean number of ICUs^a^	Medical ICU admissions ^b^			SARI admissions					
		Weekly mean (SD)	Weekly minimum and maximum	Total	Weekly mean (SD)	Weekly minimum and maximum	Total (percentage^c^)	Weekly minimum and maximum (percentage^c^)	Standardized SARI incidence^d^	SARI rate^e^
2007–2016	70	503 (145)	161–784	236,282	65 (28)	11–176	30,515 (13%)	5–25%		
2007/2008	39	239 (44)	161–316	12,411	32 (10)	11–55	1657 (13%)	6–18%	4596	3.27
2008/2009	54	343 (64)	228–494	17,851	46 (19)	14–99	2369 (13%)	5–22%	4568	3.25
2009/2010	68	434 (38)	311–502	23,023	56 (15)	25–87	2947 (13%)	7–19%	4406	3.13
2010/2011	73	485 (46)	364–601	25,222	66 (19)	33–119	3452 (14%)	7–21%	4711	3.35
2011/2012	75	523 (52)	417–658	27,188	66 (18)	36–105	3447 (13%)	8–18%	4364	3.10
2012/2013	77	574 (63)	447–735	29,862	78 (29)	35–163	4060 (14%)	8–25%	4680	3.33
2013/2014	77	608 (46)	515–733	31,620	68 (16)	39–97	3539 (11%)	7–15%	3853	2.74
2014/2015	81	667 (56)	537–784	34,682	88 (30)	46–154	4561 (13%)	8–20%	4527	3.22
2015/2016	81	649 (55)	555–744	34,423	85 (36)	39–176	4483 (13%)	6–24%	4483	3.19

*NICE* National Intensive Care Evaluation, *SARI* Severe acute respiratory infections

^a^Number of participating ICUs were calculated per calendar year (starting from 1 January)

^b^Excluding ICU admissions for surgery or trauma

^c^Percentage of total medical ICU admissions

^d^SARI admissions standardized to the total number of medical ICU admissions in season 2015/2016 (with near 100% national ICU coverage)

^e^Rate was calculated with total 15+ Dutch population size in 2015 (at a scale of 1/10,000)

#### Distribution of diagnoses in the ICU SARI-syndrome group (irrespective of influenza epidemics)

The diagnoses contributing most to SARI admissions were those registered as “Pneumonia, bacterial” (59% of SARI) and “sepsis, pulmonary” (25%) (Table 2, with a figure of weekly trends per diagnosis shown in Additional file 2) and proportions were slightly lower in 15–44-year-olds than in the older age groups. The distribution varied somewhat from season-year to season-year, most notably for “Pneumonia, viral” (1–11%) and for “Pneumonia, bacterial” (56–65%) and the proportions are shown in a table in Additional file 3. The proportion of “Pneumonia, viral” increased with decreasing age (registered in 3.5% of people age 65+ up to 8.5% of 15–44-year-olds) and “Pneumonia, aspiration” was relatively more common in the youngest group (18% of 15–44-year-olds vs 11% overall, Table 2).

**Table 2:** Proportion of SARI admissions to ICU by APACHE IV diagnosis by age group (in years) (2007–2016)

APACHE reason for admission	Age 15–44	Age 45–59	Age 60–64	Age 65+	All ages
Sepsis, pulmonary	20.51%	24.54%	25.86%	25.84%	25.11%
Pneumonia, aspiration	18.50%	11.53%	9.10%	10.39%	11.20%
Pneumonia, bacterial	49.46%	57.24%	60.36%	60.71%	58.97%
Pneumonia, fungal	0.47%	0.52%	0.47%	0.27%	0.36%
Pneumonia, other	13.00%	12.05%	12.70%	11.66%	11.98%
Pneumonia, parasitic (i,e, Pneumocystis pneumonia)	1.26%	0.77%	0.53%	0.36%	0.54%
Pneumonia, viral	8.51%	6.36%	5.12%	3.45%	4.68%

*SARI* Severe acute respiratory infections, *APACHE* Acute Physiology and Chronic Health Evaluation

#### Clinical severity: APACHE IV score and in-ICU mortality (irrespective of influenza epidemics)

While the number of ICU SARI admissions always peaked in winter, the weekly average APACHE IV score in the patients admitted was relatively stable and did not show winter peaks (Additional file 1). This was also confirmed by low correlation between the two (*R*^2^ 0.04, *p* value 0.41). Mortality in SARI within the ICU did show seasonality, with higher mortality in winter, visually coinciding with peaks in SARI admissions (*R*^2^ 0.64, *p* value <0.0001) (Fig. 1).

### ICU SARI characteristics during influenza epidemics

#### ICU SARI admissions during influenza epidemics

The standardized ICU SARI incidence during the eight influenza epidemics varied between 558 (in 2013/2014) and 2400 (in 2014/2015; the latter being the longest registered influenza epidemic in the Netherlands, lasting 20 weeks). Peak weekly incidence of ICU SARI varied by epidemic, with between 101 and 188 SARI admissions (in the 2013/2014 and 2012/2013 epidemic, respectively, standardized) or expressed as rates, 0.07–0.13/10,000 inhabitants in the peak week. The week number in which this peak occurred varied widely per epidemic (between week 45 and week 8) and the timing within the epidemic varied (occurring at the beginning, middle, or end of an influenza epidemic (Table 3). By influenza epidemic, the ICU SARI rate varied between 0.40 and 1.71 per 10,000 inhabitants.

**Table 3:** SARI admissions to adult ICU and adult ILI in sentinel GP surveillance during influenza epidemics

Season	Influenza epidemic (week numbers)^b^	Epidemic duration (weeks)	Observed SARI incidence	Standardized SARI^c^ incidence^d^	Observed ILI^a^ incidence^d^	Standardized ILI incidence^e^	Ratio of SARI^c^ vs ILI^e^	Peak SARI^f^		
								Weeknr	Incidence^cd^	Influenza strains
2007/2008	50–52 and 5–9	8	335	929	347	344	2.70	8	153	A(H1N1) dominance followed by type B dominance
2008/2009	2–6	5	379	731	476	511	1.43	2	177	A(H3) dominance
2009/2010	41–48	8	538	804	677	611	1.32	45	123	A(H1N1)pdm09
2010/2011	1–7	7	652	890	441	413	2.15	1	162	A(H1N1)pdm09 dominance followed by type B dominance
2011/2012	NA	0	NA	NA	NA	NA	NA	NA	NA	A(H3N2) dominance
2012/2013	1–16	16	1731	1995	1328	1409	1.42	6	188	Mixed A(H1N1)pdm09 and A(H3N2) dominance followed by B dominance.
2013/2014	5–8 and 10–11	6	500	544	311	329	1.65	8	101	Mixed dominance with slightly more A(H3N2) than A(H1N1)pdm09, low circulation of influenza B.
2014/2015	49–13 and 15–17	20	2386	2368	1701	1764	1.34	7	153	A(H3N2) dominance followed by influenza B
2015/2016	53–11	12	1693	1693	1103	1103	1.53	6	176	A(H1N1)pdm09 dominance followed by B dominance

*SARI* Severe acute respiratory infections, GP general practitioner, *ILI* influenza-like illness, *NA* Not applicable as there was no retrospectively registered influenza epidemic

^a^ILI in the age group of 15 years and older

^b^0 weeks crossed the ILI epidemic threshold in season 2011/2012

^c^Standardized to the total number of medical ICU admissions in season 2015/2016 (with near 100% national ICU coverage)

^d^Incidence per 10,000 inhabitants

^e^Standardized to the sentinel population size in season 2015/2016

^f^The highest registered weekly SARI incidence during an influenza epidemic

#### The relationship between ICU SARI and ILI incidence during influenza epidemics (ICU SARI/ILI ratio)

The three long epidemics of 2012/2013, 2014/2015, and 2015/2016 (12–20 weeks) showed high incidence of both ILI and of ICU SARI (Table 3). The ratio between the ICU SARI incidence and ILI incidence varies by influenza epidemic and lies between 1.3 and 2.7. This means that with every actual observed adult case of ILI in the sentinel surveillance (covering only 0.7% of the Dutch population) there were on average 1.3–2.7 observed ICU SARI admissions (covering the total adult ICU population). This ratio was lowest in 2009/2010 (the A(H1N1)pdm09 pandemic season) and highest in the 2007/2008 epidemic (Table 3). Using rates to calculate the SARI/ILI ratio, this translated to 0.008–0.017 ICU SARI admissions per estimated GP-attended ILI in the Dutch population (Table 4). In other words, per influenza epidemic, the estimated ILI rate in the total Dutch population was 59-fold to 125-fold higher than the ICU SARI rate. For example, in the 2015/2016 influenza epidemic there were 1693 adult ICU SARI admissions and an estimated total of 162,418 adult medically attended ILI cases in the total Dutch population (or 0.01 ICU SARI per estimated ILI).

**Table 4:** SARI in ICU and ILI rates in the Dutch adult population during influenza epidemics

Season	Total Dutch population size (aged 15+ years)^a^	Influenza epidemic (week numbers)	Epidemic duration (weeks)	SARI rate^b^	ILI rate^b^	SARI to ILI ratio^c^	Peak SARI^d^	
							Week number	peak SARI rate^b^
2007/2008	13,399,377	50–52 and 5–9	8	0.66	37.82	0.017	8	0.11
2008/2009	13,469,675	2–6	5	0.52	56.18	0.009	2	0.13
2009/2010	13,562,729	41–48	8	0.57	68.91	0.008	45	0.09
2010/2011	13,662,078	1–7	7	0.63	49.17	0.013	1	0.12
2011/2012	13,748,724	NA	0	NA	NA	NA	NA	NA
2012/2013	13,833,689	1–16	16	1.42	147.79	0.010	6	0.13
2013/2014	13,901,653	5–8 and 10–11	6	0.39	38.2	0.010	8	0.07
2014/2015	13,979,215	49–13 and 15–17	20	1.68	194.16	0.009	7	0.11
2015/2016	14,073,660	53–11	12	1.2	115.41	0.010	6	0.13

*SARI* Severe acute respiratory infections, GP general practitioner, *ILI* influenza-like illness, *NA* Not applicable as there was no retrospectively registered influenza epidemic

^a^Given for January–December of the first mentioned year of each season-year

^b^Rate was calculated with total 15+ Dutch population size in 2015 (at a scale of 1/10,000)

^c^Number of ICU SARI per expected medically attended ILI in the total adult Dutch population

^d^The highest registered weekly SARI incidence during an influenza epidemic

The dominant circulating influenza strain(s) varied between the epidemics with H1N1, H3N2, mixed H1N1&H3N2, and mixed domination with influenza B occurring in the study period (Table 3). The SARI incidence and the SARI/ILI ratio did not appear to depend clearly on the dominant subtype.

#### APACHE IV score during influenza epidemics

On average the mean score from the APACHE IV model (higher scores corresponding to more severe illness) varied between 71 and 78 between the epidemics and was usually higher with increasing age (Additional file 4) (note that age is factored into the score). The 2007/2008 epidemic was an exception, with the inverse being observed in the youngest three age-groups. The average APACHE IV score per influenza epidemic varied most in the youngest age group at 51–74 (2010/2011 and 2007/2008), and varied least in the 65+ age group at 77–82 (2013/2014 and 2009/2010 respectively) (see Additional file 4 for a table of these scores); the order by season mostly did not coincide in the different age groups.

#### In-ICU mortality during influenza epidemics

During the eight different influenza epidemics, the proportion of in-ICU deaths among all medical ICU admissions was 14–18%. Among ICU SARI admissions the variation was slightly greater at between 13% (2010/2011 epidemic) and 20% (2007/2008 epidemic) (Additional file 4), and mortality usually increased with increasing age except in the 2007/2008 epidemic when mortality was higher in the 15–44 year age group (16%) than in the 45–59 and 60–64 year age-groups (12%). Within the age groups, ICU SARI mortality also varied by epidemic: 2–16% in 15–44-year-olds to 16–25% in the 65+ age group. There was hardly any correlation between the proportion of ICU SARI deaths and the duration of the influenza epidemic (*R*^2^ − 0.12, *p* value 0.78), the ICU SARI incidence (standardized) during epidemics (*R*^2^ − 0.12, *p* value 0.78), and the SARI/ILI ratio (*R*^2^ − 0.11, *p* value 0.80).

## Discussion

This study helps to fill the knowledge gap on the burden of severe respiratory illness. It provides robust numbers on occurrence of ICU SARI and how these vary between the different influenza epidemics in the Netherlands. It shows how different parameters, proposed by the WHO for monitoring influenza severity [7], can vary greatly by season and that they complement each other: incidence, peak levels, ICU SARI mortality, mean APACHE IV score, and the ICU SARI/ILI ratio. They provide insight into different aspects of the severity of separate influenza seasons.

The ratio of ILI to ICU SARI admissions varied from influenza epidemic to influenza epidemic confirming that the intensive care burden of influenza is not predictable from ILI trends [25]. Depending on the epidemic, 8–17 SARI admissions occurred per 1000 cases of ILI in primary care (or a ratio of 1.3–2.7 ICU SARI admissions per each actual observed ILI in the 0.7% national ILI sentinel coverage).

Weekly medical ICU admissions were higher when SARI admissions were higher (Fig. 1) indicating that pressure on ICUs is expected to be higher in respiratory seasons. During the eight influenza epidemics under study, ICU SARI peaked at (min, max) 101 up to 188 ICU admissions in one week in the Netherlands. This is an almost twofold difference in peak-incidence between some seasons. Preparedness for severe or unusual seasons or outbreaks includes ICU capacity preparedness. The current situation in the Netherlands relies on crisis management when hospitals are to be overwhelmed. Whether and how often this occurs is not known due to lack of SARI surveillance. A SARI surveillance system might provide better insight into the location of potential bottlenecks and provide potential time gain allowing capacity planning instead of on-the-spur crisis management.

For influenza severity assessment, monitoring a SARI syndrome instead of specific diagnoses would be of use as distinguishing between bacterial and viral causes of pneumonia is difficult and bacterial pneumonia is often secondary to viral infection [26–28]. Only 4.7% of the diagnoses are registered as viral pneumonia, which may be an underestimation: laboratory data are not yet available in the NICE registry, and the specific etiology of SARI cases could not be determined. Furthermore, laboratory testing for influenza virus or other respiratory viruses is not standard practice in hospitalized patients with SARI; less than half of patients admitted to the ICU with suspected pneumonia are tested [29]. Notably in our data, in the 2015/2016 season a large proportion of ICU SARI was registered as viral. Whether this is a true finding, a registration artifact, or whether more laboratory testing occurred in that year is not clear. Further, inherent to a syndromic surveillance approach based on rapidly available provisional data, a SARI syndrome may contain some patients with an incorrect initial diagnosis at admission. Another limitation is that no data were available on influenza vaccination status or use of oseltamivir.

In terms of the highest extremes, notable influenza epidemics are those of 2007/2008, 2012/2013, and 2014/2015 for different parameters. The 2014/2015 epidemic was the longest recorded (20 weeks) and corresponded with the highest ICU SARI and ILI incidence but not with highest values of other ICU SARI characteristics. The highest peak of ICU SARI (188 in one week, standardized) was in the 2012/13 epidemic (16-week-long epidemic). In contrast, the 2007/2008 epidemic was much shorter (8 weeks) but had the highest ICU SARI/ILI ratio as was also reported in the USA [30], and the largest proportion of deaths. These results may, however, potentially have been affected by the low ICU coverage in that first study year (around 50%), although we do not know in which direction this may have impacted results.

In terms of the lowest extremes the 2009/2010 season was the pandemic season (A(H1N1)pdm09 circulation), known to have been milder in older people [31], showing the lowest ICU SARI/ILI ratio indicating a lower than average ICU burden per influenza infection in that year. The dominant virus in the following epidemic (2010/2011) was also influenza virus A(H1N1)pdm09, showing no drift to the previous season and which coincided with the smallest proportion of in -ICU SARI deaths. The 2013/2014 season was a relatively short epidemic (6 weeks) with the lowest SARI peak and with the lowest incidence of both SARI and ILI.

As the most severe outcome of any disease, mortality data, if available, could provide an important indication of flu season severity. Mortality can be high in pneumonia hospitalizations, but to date the burden of influenza mortality remains uncertain and is estimated mostly from modeling studies [13, 32]. Fatal outcome may be more prevalent in influenza-positive SARI [3]. In our study, in-ICU mortality in patients with SARI during influenza epidemics varied between 13% and 20%. To what extent this reflects differing severities of influenza seasons is not entirely clear because SARI can be caused by different respiratory pathogens, patients may die after their ICU or total hospital stay [33], and shifts in afflicted age groups can impact mortality numbers. In a German study, mortality in hospitalized patients with SARI (thus not only in the ICU) was lower (5-year-average 11%), but also included non-flu weeks [2]. Future exploration of a more comprehensive mortality measure than in-ICU deaths (i.e. 30-day mortality or survival to hospital discharge) would improve the estimate of influenza impact.

An important strength of our study is that the coverage increased over time to include almost all adult ICUs in the country, thus providing a clear estimate of the total number and incidence of ICU SARI in the Netherlands. Comparing these with other Western European countries is difficult. Most countries do not have SARI surveillance and in those that do or those that have analyzed retrospective data, the SARI case definition varies due to different coding systems and the type of hospitalizations included (ICU, other wards) [1, 2]. Additionally, criteria for ICU admission vary between countries, with Dutch ICUs being relatively restrictive. In Germany, retrospectively, ICU SARI admissions during influenza epidemics peaked at 17% [2], in the range of the weekly peak of 15–25% that we observed in the different epidemics. Of hospitalized SARI in Belgium, influenza was laboratory-diagnosed in 46%, and approximately 10% of SARI were in ICUs (sentinel surveillance of 2016/2017 epidemic) [1], similar to the 8–11% in ICUs in a Dutch pilot study in two hospitals [12] (Marbus S, et al: Acute respiratory infections in secondary care versus influenza-like illness in primary care in the Netherlands: hospital incidence peaks first, submitted). Although such estimates are not available from the national registry data (NICE) they give a rough indication that total SARI hospitalizations (when including non-ICU SARI) in the Netherlands could be around 10-fold higher than SARI in ICUs and that influenza can potentially play a large role in overall SARI admissions. Some other countries record laboratory-confirmed influenza infections in ICUs, but without tracking a denominator or testing practices, thus not providing estimates on total SARI burden [34–39].

## Conclusion

This study provides robust estimates on SARI occurrence in the Netherlands based on comprehensive national ICU registry data (NICE). Influenza severity measures proposed by the WHO vary by influenza epidemic and cannot be deduced from ILI surveillance alone, thus emphasizing the potential use of a prospective SARI surveillance for assessing the burden of seasonal influenza.

## Additional files

Additional file 1: Raw weekly numbers of admissions, APACHE IV score and mortality in adult ICUs (2007–2016). Footnote: gray shading: influenza epidemic weeks as derived from ILI sentinel surveillance data. (PDF 15 kb)

Additional file 2: Number of SARI admissions to the ICU by APACHE IV diagnosis (2007–2016). Footnote: diagnoses: sepsis, pulmonary; pneumonia, aspiration; pneumonia, bacterial; pneumonia, fungal; pneumonia, other; pneumonia, parasitic (i.e. pneumocystis pneumonia); pneumonia, viral. (PDF 18 kb)

Additional file 3: Distribution of SARI admissions to adult ICU by APACHE IV diagnosis codes per season (%). (DOCX 47 kb)

Additional file 4: In-ICU mortality and APACHE IV scores among ICU admissions during influenza epidemics. Footnote: *0 consecutive weeks crossed the ILI epidemic threshold in season 2011/2012. (DOCX 50 kb)

## Abbreviations

APACHE: Acute Physiology and Chronic Health Evaluation
ECDC: European Centre for Disease Prevention and Control
EISN: European Influenza Surveillance Network
GPs: General practitioners
ICU: Intensive care units
ILI: Influenza-like-illness
NICE: National Intensive Care Evaluation
SARI: Severe acute respiratory infections
WHO: World Health Organization
