# Supplementary figures and images for: Estimating severity of influenza epidemics from severe acute respiratory infections (SARI) in intensive care units
Source: Crit Care. 2018 Dec 19;22:351. doi: 10.1186/s13054-018-2274-8 (PMC6299979; doi:10.1186/s13054-018-2274-8)

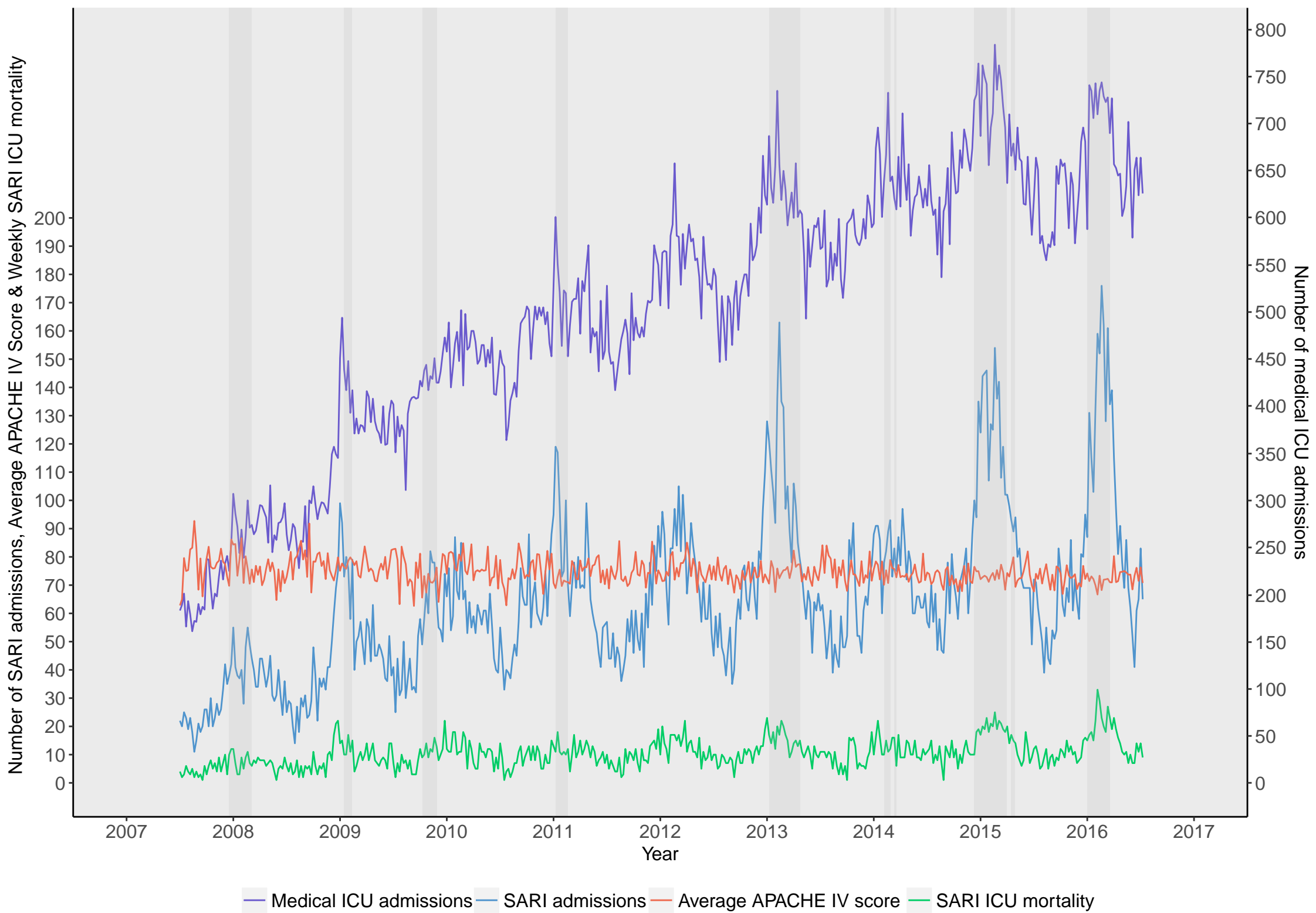

Supplement: Supplementary file 1 — Raw weekly numbers of admissions, APACHE IV score and mortality in adult ICUs (2007–2016). Footnote: gray shading: influenza epidemic weeks as derived from ILI sentinel surveillance data. (PDF 15 kb) [file 13054_2018_2274_MOESM1_ESM.pdf]

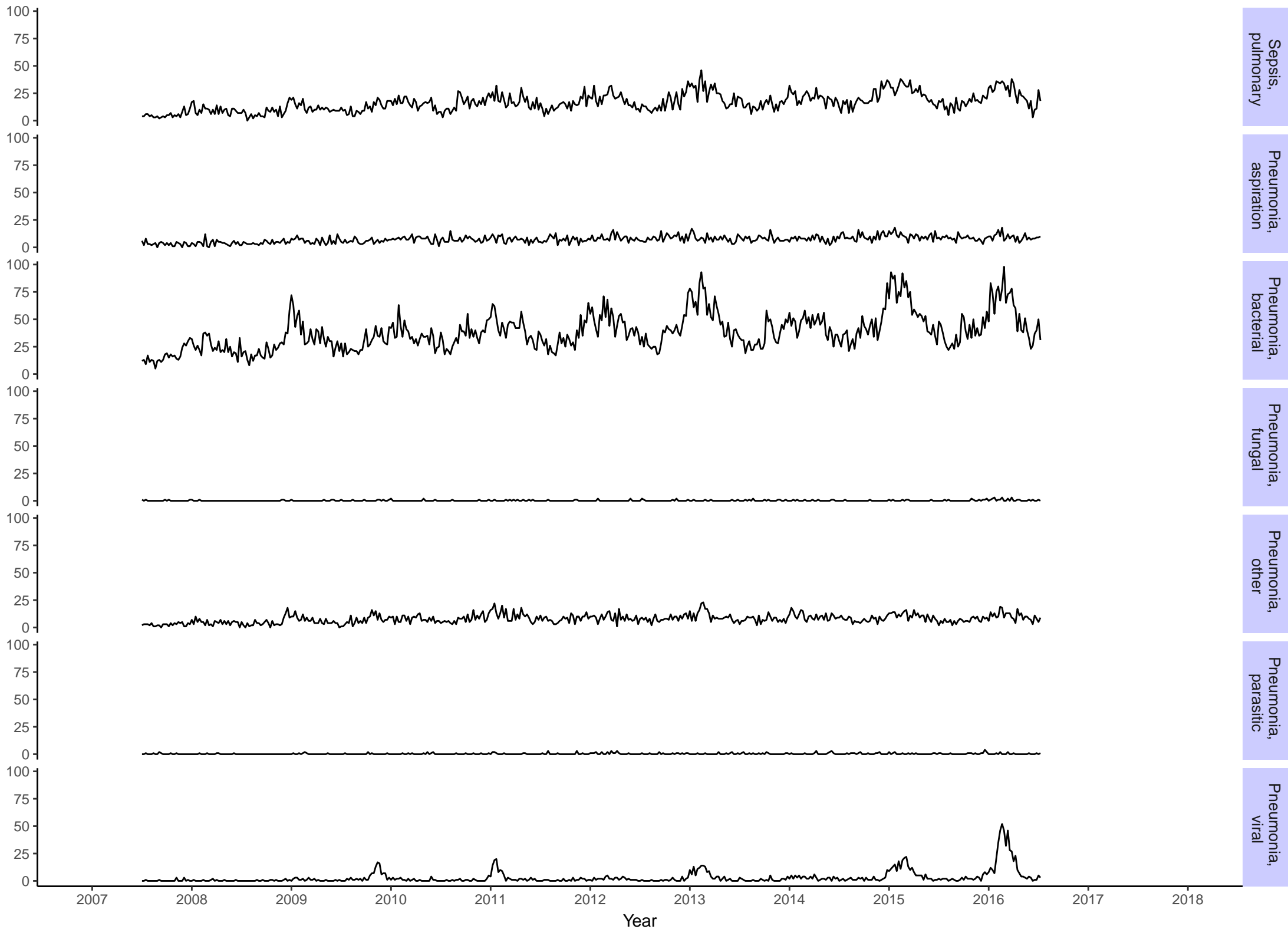

Supplement: Supplementary file 2 — Number of SARI admissions to the ICU by APACHE IV diagnosis (2007–2016). Footnote: diagnoses: sepsis, pulmonary; pneumonia, aspiration; pneumonia, bacterial; pneumonia, fungal; pneumonia, other; pneumonia, parasitic (i.e. pneumocystis pneumonia); pneumonia, viral. (PDF 18 kb) [file 13054_2018_2274_MOESM2_ESM.pdf]
