# Supplementary material for: Estimating severity of influenza epidemics from severe acute respiratory infections (SARI) in intensive care units
Source: Crit Care. 2018 Dec 19;22:351. doi: 10.1186/s13054-018-2274-8 (PMC6299979; doi:10.1186/s13054-018-2274-8)
Supplement: Supplementary file 3 — Distribution of SARI admissions to adult ICU by APACHE IV diagnosis codes per season (%). (DOCX 47 kb) [file 13054_2018_2274_MOESM3_ESM.docx]

|  | | | | | | |  |
| --- | --- | --- | --- | --- | --- | --- | --- |
|  |  |  |  |  |  |  |  |
| Season | Sepsis, pulmonary | Pneumonia, aspiration | Pneumonia, bacterial | Pneumonia, fungal | Pneumonia, other | Pneumonia, parasitic | Pneumonia, viral |
| 2007/2008 | 22.93% | 10.92% | 65.30% | 0.42% | 11.47% | 0.72% | 1.03% |
| 2008/2009 | 21.19% | 11.44% | 64.84% | 0.25% | 12.07% | 0.30% | 1.39% |
| 2009/2010 | 24.09% | 11.98% | 57.38% | 0.41% | 13.20% | 0.48% | 4.51% |
| 2010/2011 | 25.58% | 10.89% | 57.65% | 0.32% | 14.34% | 0.38% | 3.74% |
| 2011/2012 | 25.70% | 11.72% | 60.05% | 0.20% | 12.50% | 0.81% | 2.00% |
| 2012/2013 | 26.50% | 10.81% | 59.09% | 0.30% | 12.07% | 0.49% | 3.99% |
| 2013/2014 | 26.19% | 11.27% | 57.81% | 0.45% | 13.08% | 0.71% | 2.68% |
| 2014/2015 | 26.29% | 11.58% | 58.65% | 0.18% | 10.13% | 0.53% | 6.05% |
| 2015/2016 | 24.49% | 10.42% | 55.88% | 0.69% | 10.06% | 0.51% | 11.47% |
| Rows add up to more than 100% due to some patients having two different diagnoses codes. | | | | | | |  |
|  |  |  |  |  |  |  |  |
