# Supplementary material for: Estimating severity of influenza epidemics from severe acute respiratory infections (SARI) in intensive care units
Source: Crit Care. 2018 Dec 19;22:351. doi: 10.1186/s13054-018-2274-8 (PMC6299979; doi:10.1186/s13054-018-2274-8)
Supplement: Supplementary file 4 — In-ICU mortality and APACHE IV scores among ICU admissions during influenza epidemics. Footnote: *0 consecutive weeks crossed the ILI epidemic threshold in season 2011/2012. (DOCX 50 kb) [file 13054_2018_2274_MOESM4_ESM.docx]

|  | | | | | | | | | | | | | | |  |  | |  |  | |  |  |
| --- | --- | --- | --- | --- | --- | --- | --- | --- | --- | --- | --- | --- | --- | --- | --- | --- | --- | --- | --- | --- | --- | --- |
| Season | Influenza epidemic (weeknr)* | In-ICU Deaths among all medical ICU admissions | Total SARI in-ICU deaths |  | In-ICU Deaths among SARI admissions | | | | |  | Mean APACHE IV Score of SARI admissions | | | | | | | | |  |  |  |
|  |  |  |  |  | Age 15-44 | Age 45-59 | Age 60-64 | Age 65+ | **All ages** |  | Age 15-44 | Age 45-59 | Age 60-64 | Age 65+ | | | **All ages** | | |  |  |  |
| 2007/2008 | 50-52 & 5-9 | 18% | 66 |  | 16% | 12% | 12% | 25% | **20%** |  | 74 | 71 | 70 | 80 | | | **77** | | |  |  |  |
| 2008/2009 | 2-6 | 17% | 63 |  | 14% | 16% | 16% | 17% | **17%** |  | 69 | 74 | 82 | 80 | | | **78** | | |  |  |  |
| 2009/2010 | 41-48 | 16% | 94 |  | 6% | 12% | 16% | 24% | **17%** |  | 56 | 61 | 70 | 82 | | | **71** | | |  |  |  |
| 2010/2011 | 1-7 | 14% | 83 |  | 5% | 10% | 16% | 16% | **13%** |  | 51 | 69 | 75 | 79 | | | **72** | | |  |  |  |
| 2011/2012 | 0 | NA | NA |  | NA | NA | NA | NA | **NA** |  | NA | NA | NA | NA | | | **NA** | | |  |  |  |
| 2012/2013 | 1-16 | 14% | 249 |  | 5% | 9% | 14% | 18% | **14%** |  | 58 | 68 | 78 | 80 | | | **75** | | |  |  |  |
| 2013/2014 | 5-8 & 10-11 | 14% | 70 |  | 2% | 11% | 14% | 17% | **14%** |  | 52 | 70 | 75 | 77 | | | **74** | | |  |  |  |
| 2014/2015 | 49-13 & 15-17 | 14% | 375 |  | 9% | 7% | 13% | 19% | **16%** |  | 52 | 63 | 74 | 79 | | | **74** | | |  |  |  |
| 2015/2016 | 53-11 | 14% | 264 |  | 7% | 11% | 12% | 20% | **16%** |  | 57 | 64 | 69 | 78 | | | **71** | | |  |  |  |
| **2007 - 2016** | **NA** | **14%** | **1264** |  | **7%** | **10%** | **14%** | **19%** | **15%** |  | **58** | **66** | **74** | **79** | | | **74** | | |  |  |  |
| *0 consecutive weeks crossed the ILI epidemic threshold in season 2011/2012 | | | | | | |  |  |  |  |  |  |  |  | | |  | | |  |  |  |
